# Acetyl-L-Carnitine Modulates TP53 and IL10 Gene Expression Induced by 3-NPA Evoked Toxicity in PC12 Cells

**DOI:** 10.2174/157015911795017182

**Published:** 2011-03

**Authors:** A Virmani, A Koverech, S.F Ali, Z.K Binienda

**Affiliations:** a Scientific & Medical Affairs, Sigma Tau SpA, Pomezia, 00040, Roma, Italy; b Neurochemistry Laboratory, Division of Neurotoxicology, NCTR/FDA, Jefferson, AR, USA; c Neurophysiology Laboratory, Division of Neurotoxicology, NCTR/FDA, Jefferson, AR, USA

**Keywords:** 3-Nitropropionic acid, Acetyl-L-carnitine, Neuroprotection, Gene expression, TP53, Il10.

## Abstract

The neurotoxicity induced by the mitochondrial inhibitor 3-nitropropionic acid (3-NPA) is associated with a decrease of ATP synthesis and an increase of free radical production which can lead to apoptosis or necrosis. We have used the PC12, neuron-like rat pheochromocytoma cell line, to study further the mechanism of 3-NPA-evoked neurotoxicity and the effects of acetyl-L-carnitine (ALC) which has neuroprotective actions against various types of mitochondrial inhibitors.

Cultured PC 12 cells were exposed to a low dose of 3-NPA 50 (microM) in the presence or absence of 5 mM ALC. The dose of 3-NPA was sub toxic and no changes in pro-apoptotic Bax or anti-apoptotic Bcl-2 gene expression were observed. We followed specific genetic markers to look for changes evoked by 3-NPA toxicity and also changes associated with neuroprotection exerted by the ALC treatment, using RT-PCR arrays (delta-delta method). 3-NPA exposure evoked a decrease in expression of the Tp53 gene. This down regulation was prevented by pretreatment of the cells with ALC. The Tp53 gene responds to cellular stresses and the effects seen here are possibly associated with the 3-NPA evoked changes in mitochondrial metabolism. Other genes associated with stress and apoptosis, Parp-1, Bcl-2, and Bax were not affected by 3-NPA or ALC. The decrease of inflammatory response Il-10 gene expression due to 3-NPA was further lowered by presence of ALC. Other inflammation related genes, Il1rn, Nr3c1 and Cxcr4 were not affected. Interestingly, the glutamate transporter slc17a7, carnitine-acylcarnitine translocase Slc25a20 and heat shock proteins genes, Hsp27, Hmox1 (Hsp32, HO1) as well as Hspa 1a (Hsp 70) increased only when both ALC and small dose of 3-NPA were present. The alterations in gene expression detected in this study suggest role of several intracellular pathways in the neurotoxicity of 3-NPA and the neuroprotection against 3-NPA-induced neurotoxicity by ALC.

## INTRODUCTION

The mitochondrial inhibitor, 3-nitropropionic acid (3-NPA), induces cellular energy deficit through inactivation of electron transport chain complex II (succinate dehydrogenase). A decrease of ATP synthesis and an increase of free radical production followed by apoptosis or necrosis caused by secondary excitotoxicity are major features of 3-NPA generated neurotoxicity [1]. These changes are associated with a decrease of body temperature and a decrease of dopamine production in susceptible brain regions in animal model of 3-NPA neurotoxicity [2, 3]. 3-NPA causes activation of NMDA receptors in dopaminergic, GABAergic and glutamatergic systems [1-4].

The neuron-like rat pheochromocytoma cell line, PC12, has been used to study mechanism of neurotoxicity and neuroprotection in dopaminergic neurons [5, 6]. The single millimolar dose of 3-NPA results in apoptotic and necrotic death of PC12 cells [7]. The application of energy metabolism enhancers such as acetyl-L-carnitine (ALC) or coenzyme Q10, have been reported to prevent this neurotoxicity [8-12]. Endogenous L-carnitine is physiologically required for the transfer of free fatty acids across the mitochondrial membrane for β-oxidation but may also have a number of other functions ranging from osmolarity to effects on cellular membrane [13].

Our earlier studies have focused on finding genetic markers of 3-NPA toxicity [14] and the possible genetic changes associated with neuroprotection by metabolic modifiers such as carnitines [9]. We reported previously an activation of uncoupling protein-2 gene in the rat striatum along with genes related to dopamine metabolism in response to 3-NPA, which was attenuated by L-carnitine pretreatment [8]. The present study was designed to investigate an effect of sub toxic doses of 3-NPA on expression of selected genes, representing pathways involved in metabolism of carnitine, glutamate transport, apoptosis, heat shock, and inflammatory responses. The neuroprotective actions of ALC were investigated as well.

## MATERIAL AND METHODS

### PC12 Culturing, Treatment and Harvest

The PC12 cell line (Pheochromocytoma cell line) was obtained from the American Type Culture Collection (ATCC, Rockville, MD). The undifferentiated PC12 cells were grown in RPMI medium supplemented with 10% fetal bovine serum (Hyclone, Logan, USA). Cells were counted and seeded in 12 well dishes at concentration 0.75 x 10^6^ cells per well in 2 ml medium. Cells were then treated with 50 microM 3-NPA, 5 mM ALC or with combination of both and incubated for 24 hrs. After withdrawal of the media cells were washed with 1 ml of HBSS, followed by aspiration of the media and further incubation in 0.5 ml of trypsin solution for 2 min. 10 ml of RPMI-1640 was added, and solution was centrifuged. Cell pellets were frozen immediately in -80°C.

### RNA Isolation and Real-time RT-PCR

Total RNA from each frozen cell pellet was isolated using RNAqueous - 4PCR, Ambion, Austin, TX. The integrity and purity of total RNA was determined with the RNA 6000LabChip kit and Agilent 2100 Bioanalyzer (Agilent Technologies, Wilmington, DE) [14]. For each sample, cDNA was synthesized with random hexamers and 2 ug of total RNA using an iScript cDNA Synthesis Kit (Bio-Rad Laboratories, Inc., Hercules, CA). The cDNA was aliquoted and stored at -80° C until use.

PCR primer sequences were designed using the NCBI genes database and the Taqman Probe and Primer Design function (ignoring the probe) of the Primer Express v 1.5 software of Applied Biosystems (Applied Biosystems, Foster City, CA). All primers were synthesized by IDT, Inc (Coralville, IA). During the first step of analysis the samples in each of the 4 experimental groups were combined and C_T_ was estimated using the 2-ΔΔ*C*_T_ method [15]. This screen allowed selecting 16 pair of primers representing genes which transcripts are most likely being altered by the treatments.

Table **1** contains sequences of 16 pairs of primers used for further quantification of transcripts. All real time PCR reactions were carried out using 2xSYBR Green Master Mix (iTaq SYBR Green Supermix with ROX, Bio-Rad Laboratories, Inc., Hercules, CA) and the ABI Prism 7700 Sequence Detection System. Assay parameters (Table **2**) were optimized and relative gene expression was calculated using standard curves (log of the ng RNA-equivalents of cDNA versus cycle number) generated using 4-fold serial dilutions of cDNA. The cDNA was a pool of all cDNA’s prepared in this experiment. Data were normalized to 18 s ribosomal RNA. Values of expression presented in results figures are ng of each gene of interest per ng of 18 S relative to a standard curve for each gene of interest.

### Data Analysis

Nonparametric (Kruskal-Wallis Rank Sum Test) and parametric (*t-*Test) statistical tests were applied using SigmaStat statistical package (Systat Software, Inc. Richmond, CA). Values of p<0.05 were considered statistically significant.

## RESULTS

ALC alone did not cause changes in genes expression (the 16 genes tested) in the PC12 cell culture. The significant differences in several genes expressions were observed between the control and 3-NPA and between control and 3-NPA pretreated with ALC. The changes in the expression of the genes by the PC12 cells in response to 3-NPA alone or in the presence of the 3-NPA and ALC are summarized in Table **3**.

The decrease in Tp53 gene expression in response to the 3-NPA (50 microM) was abolished by the pretreatment of the PC12 cells with ALC (5 mM) (Table **3**). In contrast the decrease in IL-10 gene expression in cells exposed to 3-NPA was further lowered by ALC “Fig. (**1**)”. The 3-NPA provoked a significant increase in Ptgs1 (Cox1) gene expression which was increased by the presence of ALC but not significantly “Fig. (**2**)”. Other genes related to inflammation, Il1rn, Nr3c1 and Cxcr4 were not affected by either 3-NPA or 3-NPA plus ALC. Interestingly, the glutamate transporter slc17a7, carnitine-acylcarnitine translocase Slc25a20 and heat shock proteins genes, Hsp27, Hmox1 (Hsp32, HO1) and Hspa 1a (Hsp 70) increased only when both ALC and small dose of 3-NPA were present.

## DISCUSSION

The plant and fungal toxin, 3-NPA, acts as an inhibitor of mitochondrial function *via* irreversible inactivation of the mitochondrial inner membrane enzyme, succinate dehydrogenase (SDH) [16]. Inhibition of SDH disturbs electron transport and leads to cellular energy deficits and neuronal injury. In the present study, we examined the effect of low 3-NPA dose on expression of panel of selected genes, representing pathways involved in metabolism of carnitine, glutamate transport, apoptosis, heat shock proteins and inflammatory responses. Our specific objective was to identify genetic markers of neurotoxicity and neuroprotection which could eventually lead to discovery of new function or pathways for “old” genes and proteins. Cells were treated with 50 µM 3-NPA, 5 mM acetyl-L-carnitine (ALC) or combination of 3-NPA and ALC and incubated for 24 hrs.

We observed a decrease of Tp53 gene expression, which was abolished by pretreatment of the cells with ALC. The initiation of apoptosis is usually associated with an increase in the expression of Tp53. Activated Tp53 can translocate into nucleus and activate proapoptotic Bax. The decrease of expression of Tp53 might be related to other functions of this protein. The main function of Tp53 is to maintain genomic stability in the cell.

In genetic pathways of excitotoxicity, a decrease of Tp53 activity can delay Ca^2++^ induced apoptosis. Another mechanism through which Tp53 could regulate apoptosis may involve NAD^+^-dependent histone deacetylases. The association of human deacetylases SIRT1 with Tp53 results in deacetylation of Tp53. This further inhibits transcriptional activity of Tp53 and prevents apoptosis. SIR1 mediated deacetylation of Tp53 may lead to cell survival under metabolic stress condition. The NAD^+^ and histone deacetylases mediated anti-apoptotic action of Tp53 remains to be explored in the study of neuroprotective agents such as ALC.

A decrease of Il-10 gene expression after PC12 exposure to 3-NPA was further lowered by presence of ALC. The Il-10 anti-inflammatory pathway plays an important role in the central nervous system by downregulating production of microglial proinflammatory signals. In the classical Il-10 antiinflammatory signaling pathway, Il-10 interacts with Il-10 receptor, which belongs to JAK/STAT class of receptors. Down regulation of Il-10 pathway found in PC12 cells (non-inflammatory cell line) did not cause the downregulation of HO-1. This indicates possibility of other housekeeping function of Il-10 and activation of transcriptional regulators of HO-1 different from Il-10. There is also an intriguing possibility of co-regulation of Cox1 and Il-10 which both are involved in hypothermia regulation. In a mammal model of hypothermia, Il-10 secures normothermia. Hypothermia is a physiological marker in 3-NPA animal models of neurotoxicity [3].

Interestingly, the glutamate transporter vesicular glutamate transporter (Slc17a7 gene), the carnitine-acylcarnitine translocase (Slc25a20) and the heat shock proteins genes (Hsp27, Hsp 32, Hsp70) increased only when both ALC and small dose of 3-NPA were present. The increase of transcription of vesicular glutamate transporter gene may represent protective response to metabolic stress. The increase in expression of the acyl-carnitine –carnitine exchanger would have effects on mitochondrial metabolism and the role of the increased expression of both Slc17a7 and Slc25a20 genes in the possible neuroprotective action against 3-NPA needs to be studied further. With regards to the heat shock proteins, they have been implicated in multiple functions in cellular stress conditions.

In summary, the gene expression alterations seen in the present study suggest role of several intracellular pathways in the neurotoxicity of 3-NPA and the neuroprotection by ALC. These changes probably reflect the “remodeling” of gene expression to maintain cell energy status [17]. Further *in vivo* studies are necessary to compare the sub-lethal and lethal doses of the 3-NPA and the effects of other putative neuroprotective agents.

## Figures and Tables

**Fig. (1):**
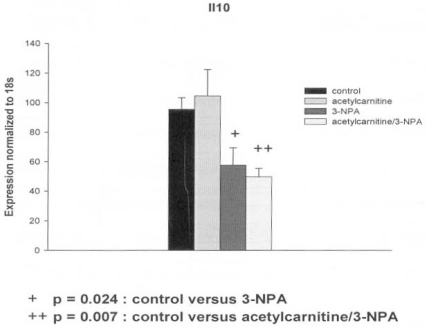
Effect of 3-nitropropionic acid (3-NPA) and acetyl-Lcarnitine (ALC) on Il10 gene expression in PC12 cells. Mean ± SEM.

**Fig. (2):**
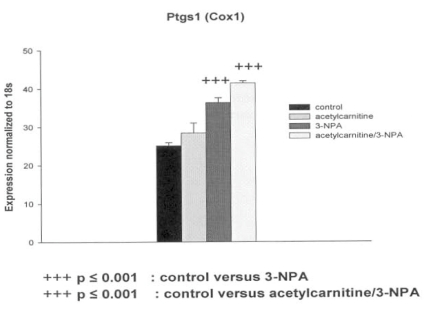
Effect of 3-nitropropionic acid (3-NPA) and acetyl-Lcarnitine (ALC) on Ptgs1 (Cox1) gene expression in PC12 cells. Mean ± SEM.

**Table 1:** Genes Analyzed in PC12 Cells

Symbol	Accession #	Description
***Apoptosis***		
Parp-1	NM_013063	poly(ADP-ribose) polymerase family, member 1
Tp53	NM_030989	tumor protein p53
Pdcd8 (AIF)	NM_031356	programmed cell death 8
Bcl2	NM_016993	B-cell leukemia/lymphoma 2
Bax	NM_017059	Bcl2-associated X protein
***Inflammation***		
Il10	NM_012854	interleukin 10
Il1rn	NM_022194	interleukin 1 receptor antagonist
Nr3c1	NM_012576	nuclear receptor subfamily 3, group C, member 1
Cxcr4	NM_022205	chemokine (C-X-C motif) receptor 4
Ptgs1 (Cox1)	NM_017043	prostaglandin-endoperoxidase synthase 1
***Transporters***		
Slc17a7	NM_053859	solute carrier family 17 (sodium-dependent inorganic phosphate cotransporter), member 7
Slc25a20	NM_053965	solute carrier family 25 (mitochondrial carnitine/acylcarnitine translocase), member 20
Slc25a14 (ucp5)	NM_053501	solute carrier family 25 (mitochondrial carrier, brain), member 14
***Heat Shock Proteins***		
Hsp27	M86389	heat shock protein 27
Hmox1 (Hsp27,HO1)	NM_012580	heme oxygenase (decycling) 1
Hspa 1a (Hsp70)	NM_031971	heat shock 70 kDa protein 1A

**Table 2:** Primers Used in Real Time RT-PCR SYBR Green Assay to Quantify Genes Expression in PC12 Cells

Symbol	Accession #	5' Primer	3' Primer
Parp-1	NM_013063	cctgacccttcggccag	caccagacggaatccctgtt
Tp53	NM_030989	catgagcgttgctctgatgg	gatttccttccacccggataa
Pdcd8	NM_031356	tgcgtccagaggccga	acgccattgctggaag
Bcl2	NM_016993	gggacgctttgccacg	cacaatcctcccccagttca
Bax	NM_017059	tccgtgtggcagctgacat	aagggcaaccacccgg
Il10	NM_012854	tgcaacagctcagcgca	gtcacagctttcgagactggaa
Il1rn	NM_022194	gcgctttaccttcatccgc	ctggacaggcaagtgattcga
Nr3c1	NM_012576	gggaccacctcccaagct	caccccgtaatgacatcctga
Cxcr4	NM_022205	ctgtggatggtggtgttcca	acgatgcccggcagg
Ptgs1	NM_017043	gcccagttccagtatcgca	gcggatgccagtgatagagg
Slc17a7	NM_053859	ccaatgtgcgaaagctgatgaa	acgcccttggagtgtgagta
Slc25a20 cac	NM_053965	ccccttggacacggtcaa	ggtggctgcccaggc
Slc25a14 ucp5	NM_053501	gccatgagatgtctggtctgaa	aactcggcaacaatagaggca
Hsp27	M86389	cgatcgctggcgcg	ggtcttaactgtgagctcctcagg
Hmox1	NM_012580	cgaaacaagcagaacccagtc	gcagcccttcggtgca
Hspa 1a	NM_031971	caagaatgcgctcgagtcct	gctgatcttgcccttgagacc

**Table 3:** Effects of 3-NPA with and without Acetyl-L-Carnitine Pretreatment on Gene Expression in PC12 Cells

GENES	3-NPA		Acetylcarnitine/3-NPA	
	Change	P values	Change	P values
***Apoptosis***				
Parp-1				
Tp53	down	p = 0.009		
Pdcd8 (AIF)			up	p = 0.05
Bcl2				
Bax				
***Inflammation***				
Il10	down	p = 0.024	down	p = 0.007
Il1rn				
Nr3c1				
Cxcr4				
Ptgs1 (Cox1)	up	p ≤ 0.001	up	p ≤ 0.001
***Transporters***				
Slc17a7			up	p ≤ 0.001
Slc25a20			up	p = 0.018
Slc25a14 (ucp5)				
***Heat Shock Proteins***				
Hsp27			up	p ≤ 0.001
Hmox1 ( Hsp32, HO1)			up	p = 0.024
Hspa 1a (Hsp70)			up	p = 0.009

## References

[1] Pang Z, Geddes JW (1997). Mechanisms of cell death induced by the mitochondrial toxin 3-nitropropionic acid: Acute excitotoxic necrosis and delayed apoptosis. J. Neurosci.

[2] Brouillet E, Jenkins BG, Hyman BT, Ferrante RJ, Kowall NW, Srivastava R, Roy DS, Rosen BR, Beal MF (1993). Age-dependent vulnerability of the striatum to the mitochondrial toxin 3-nitropropionic acid. J. Neurochem.

[3] Nony PA, Scallet AC, Rountree RL, Ye X, Binienda Z (1999). 3-Nitropropionic acid (3-NPA) produces hypothermia and inhibits histochemical labelling of succinate dehydrogenase (SDH) in rat brain. Metab. Brain Dis.

[4] Zeevalk GD, Derr-Yellin E, Nicklas WJ (1995). Relative vulnerability of dopamine and GABA neurons in mesencephalic culture to inhibition of succinate dehydrogenase by malonate and 3-nitropropionic acid and protection by NMDA receptor blockade. J. Pharmacol. Exp. Ther.

[5] Virmani A, Gaetani F, Binienda Z, Xu A, Duhart H, Ali SF (2004). Role of mitochondrial dysfunction in neurotoxicity of MPP^+^: partial protection of PC12 cells by acetyl-L-carnitine. Ann. N. Y. Acad. Sci.

[6] Wang J, Rahman MF, Duhart HM, Newport GD, Patterson TA, Murdock RC, Hussain SM, Schlager JJ, Ali SF (2009). Expression changes of dopaminergic system-related genes in PC12 cells induced by manganese, silver, or copper nanoparticles. Neurotoxicology.

[7] Mandavilli BS, Boldogh I, Van Houten B (2005). 3-nitropropionic acid-induced hydrogen peroxide, mitochondrial DNA damage, and cell death are attenuated by Bcl-2 overexpression in PC12 cells. Brain Res. Mol. Brain Res.

[8] Binienda ZK, Ali SF, Virmani A, Amato A, Salem N, Przybyla BD (2006). Co-regulation of dopamine D1 receptor and uncoupling protein-2 expression in 3-nitropropionic acid-induced neurotoxicity: neuroprotective role of L-carnitine. Neurosci. Lett.

[9] Binienda ZK, Przybyla BD, Robinson BL, Salem N, Virmani A, Amato A, Ali SF (2006). Effects of L-carnitine pretreatment in methamphetamine and 3-nitropropionic acid-induced neurotoxicity. Ann. NY Acad. Sci.

[10] Virmani MA, Biselli R, Spadoni A, Rossi S, Corsico N, Calvani M, Fattorossi A, De Simone C, Arrigoni-Martelli E (1995). Protective actions of L-carnitine and acetyl-L-carnitine on the neurotoxicity evoked by mitochondrial uncoupling or inhibitors. Pharmacol. Res.

[11] Virmani A, Gaetani F, Binienda Z (2005). Effects of metabolic modifiers such as carnitines, coenzyme Q10, and PUFAs against different forms of neurotoxic insults: metabolic inhibitors, MPTP, and methamphetamine. Ann. N.Y. Acad. Sci.

[12] Binienda Z, Przybyla-Zawislak B, Virmani A, Schmued L (2005). L-carnitine and neuroprotection in the animal model of mitochondrial dysfunction. Ann. N. Y. Acad. Sci.

[13] Virmani A, Binienda Z (2004). Role of carnitine esters in brain neuropathology. Mol. Aspects Med.

[14] Przybyla-Zawislak BD, Thorn BT, Ali SF, Dennis RA, Amato A, Virmani A, Binienda ZK (2005). Identification of rat hippocampal mRNAs altered by the mitochondrial toxicant, 3-NPA. Ann. N.Y. Acad. Sci.

[15] Livak KJ, Schmittgen TD (2001). Analysis of relative gene expression data using real-time quantitative PCR and the 2(-Delta Delta C(T)) Method. Methods.

[16] Coles CJ, Edmondson DE, and Singer TP (1979). Inactivation of succinate dehydrogenase by 3-nitropropionic acid. J. Biol. Chem.

[17] Brambrink AM, Schneider A, Noga H, Astheimer A, Götz B, Körner I, Heimann A, Welschof M, Kempski O (2000). Tolerance-Inducing dose of 3-nitropropionic acid modulates bcl-2 and bax balance in the rat brain: a potential mechanism of chemical preconditioning. J. Cereb. Blood Flow Metab.

